# Mononuclear phagocyte sub-types in vitro display diverse transcriptional responses to dust mite exposure

**DOI:** 10.1038/s41598-024-64783-1

**Published:** 2024-06-20

**Authors:** Leonie F. H. Fransen, Martin O. Leonard

**Affiliations:** Toxicology Department, Radiation, Chemical and Environmental Hazards Directorate, UK Health Security Agency, Harwell Science and Innovation Campus, Harwell, OX11 0RQ UK

**Keywords:** Mononuclear phagocytes, Myeloid, CD34, RNA-seq, Diesel exhaust particles, Dust mite, Asthma, Mucosal immunology

## Abstract

Mononuclear phagocytes (MNP), including macrophages and dendritic cells form an essential component of primary responses to environmental hazards and toxic exposures. This is particularly important in disease conditions such as asthma and allergic airway disease, where many different cell types are present. In this study, we differentiated CD34+ haematopoietic stem cells towards different populations of MNP in an effort to understand how different cell subtypes present in inflammatory disease microenvironments respond to the common allergen house dust mite (HDM). Using single cell mRNA sequencing, we demonstrate that macrophage subtypes MC^SPP1+^ and MLC^MARCO+^ display different patterns of gene expression after HDM challenge, noted especially for the chemokines CXCL5, CXCL8, CCL5 and CCL15. MLC^CD206Hi^ alternatively activated macrophages displayed the greatest changes in expression, while neutrophil and monocyte populations did not respond. Further work investigated how pollutant diesel exhaust particles could modify these transcriptional responses and revealed that CXC but not CC type chemokines were further upregulated. Through the use of diesel particles with adsorbed material removed, we suggest that soluble pollutants on these particles are the active constituents responsible for the modifying effects on HDM. This study highlights that environmental exposures may influence tissue responses dependent on which MNP cell type is present, and that these should be considerations when modelling such events in vitro. Understanding the nuanced responsiveness of different immune cell types to allergen and pollutant exposure also contributes to a better understanding of how these exposures influence the development and exacerbation of human disease.

## Introduction

Mononuclear phagocytes (MNPs) including monocyte, macrophage and dendritic cells are cells of myeloid origin and play important roles in maintaining tissue homeostasis and defence against injury and infection^1,2^. Within the lung, these cells have central roles in directing innate and adaptive immunological processes in conditions such as allergy and asthma^3,4^. From the early stages of disease development right through to established inflammatory environments within the lung, MNPs continuously respond to inhalation exposures, propagating often unwanted effects to the detriment of organ function^5,6^. In addition to airway epithelial cells, resident populations of innate immune cells, such as interstitial and alveolar macrophages have been identified as driving the initial responses to environmental exposures such as house dust mite (HDM)^7,8^. The receptor Dectin-2 specifically expressed on alveolar macrophages was identified as critical for initial responses to HDM but not for the propagation of inflammatory disease^8^. Indeed, the absence of alveolar macrophages during these exposures is associated with a worsening of allergic airway inflammation^7,9^. While much has been revealed regarding the role of migrating inflammatory dendritic cells in driving adaptive immunity from allergen exposures^10^, much less is known regarding inflammatory macrophage responses to environmental exposures and their role in inflammatory lung disease. One recent study in the mouse lung has however identified that Ccr2+ monocyte derived cd11c+ macrophages drive HDM induced allergic inflammation^11^. This lack of understanding of MNP responses in active inflammatory disease becomes ever more striking, when new studies reveal the diversity of cell types present in these conditions^12^.

In addition to HDM allergen exposure, the effect of combustion pollutant particles such as diesel exhaust particles (DEP) within the environment is also a concern for human health. It has also been documented that these particles can have adjuvant and exacerbation effects on asthmatic and allergen driven lung disease^13–17^. How these exposure types interact, and the mechanisms involved in worsening of lung disease is still unclear. Importantly, there is a well-documented increase in MNPs within the airways after exposure to DEP and HDM^13–16^. Much of the work to determine the responses to pollutant particles and dust mite allergens, including their interaction has been focussed to investigating dendritic, epithelial and neutrophilic cell types^18–20^ with much less focus on the role of macrophage subtypes.

With the advent of new techniques such as single cell sequencing (ScSeq), allowing detailed cellular and molecular analyses of complex disease states, it is becoming increasingly evident that an even larger number of cell types and cell states exist than previously appreciated, all with their own specific functions and potential for unique responses to external stimuli. For example, ScSeq analysis of endobronchial brushings from allergic and asthmatic patients revealed up to 14 different types of MNPs^12^. Some types of MNPs have been specifically implicated in the pathogenesis of allergic asthma, for example Cd11c expressing phagocytes^3^ or macrophages expressing high levels of CD206 among the often termed alternatively activated macrophages (AAM). These latter cells have been demonstrated as a contributor to asthmatic airway disease in mouse models of allergy and are also present at higher numbers in human asthmatic airways^21^. AAM typically differentiate within type 2 immune environments observed in allergic inflammation, through the action of cytokines such as IL-4 and IL-13 derived from ILC2 or T_H_2 T-cell mediated adaptive immune responses^22^. However, there are many other MNPs, which are less well characterised, particularly in their ability to directly respond to environmental exposures and how they may contribute to disease exacerbation and development within the lung.

In this study, we used human primary bone marrow derived CD34+ stem cells to generate different populations of MNPs, found within inflammatory airway disease, with the aim to test responses to pollutant particulate and allergen exposure^23,24^. These stem cells give rise to many if not all of the infiltrating MNP cells in allergic and asthmatic lung disease and have also been observed to replace alveolar macrophages^25^ as they are depleted from the lung in disease. We therefore considered this in vitro model an appropriate test system to establish a diverse array of MNPs from a primary cell source to best capture the inflammatory cell type responses to environmental adverse exposures as would occur during active lung inflammatory disease. Our previous work has shown, using a protocol specific for macrophages like cell (MLC) differentiation, overexpression of markers reflective of different macrophage cell types, including SPP1, MARCO and CD206, and similarly for dendritic cell (DC) types using a DC specific protocol^23^. Building from this work in our current study, we have used Scseq to further characterise the different cell types within these in vitro systems and tested how some of them respond to HDM exposure. Again, building on previous work, where we demonstrated that CD34+ derived macrophages respond to particle and house dust mite exposures through an increase in inflammatory mediators including CXCL5, CCL15 and metallothionein MT1M expression^24^, our aim in this study was to identify the cell types responsible for these changes in gene expression. Ultimately, through the identification of MNP cell types, which are highly responsive to environmental stimuli, and present within diseased airways, one can help refine mechanistic understanding of how inhaled exposures contribute to exacerbation of disease conditions such as allergic asthma. This information is also important for how to best model these responder events in order to uncover which molecular components of environmental toxins and allergens exist, as well as their interactions that can drive allergic airway disease.

## Methods

### CD34+ haematopoietic stem cell culture and myeloid differentiation

**Figure 1:**
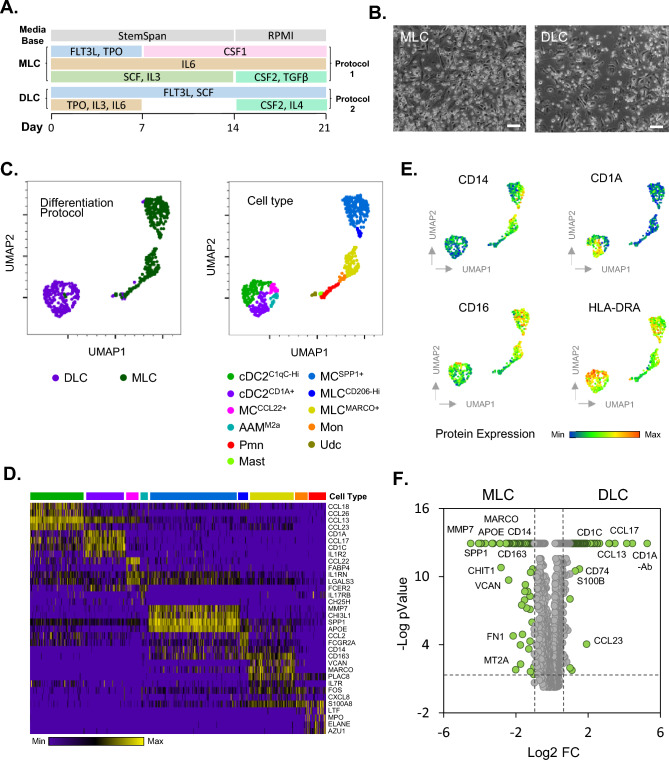
CD34+ Hematopoietic stem cell derived myeloid cell differentiation and characterisation. Human primary bone marrow derived CD34+ cells were differentiated along a myeloid trajectory towards macrophage like (MLC) and dendritic like (DLC) cell phenotypes as indicated (**A**), including morphological assessment (Scale bar; 50 µm) (**B**). Single cell RNA-Seq characterisation of cell populations revealed distinct cell types visualised using UMAP dimensional reduction (**C**). Cell types included conventional dendritic cells (cDC2), macrophages (MC), alternatively activated macrophages (AAM), neutrophils (PMN), monocytes (Mon) and undefined cells (Udc). ScSeq defined cell type uniquely expressed marker genes are visualised as a heatmap (**D**) with cell type colour key indicated in (**C**). Surface protein levels for immune cell markers was carried out using Ab-seq labelling (**E**). MLC and DLC whole population Scseq mRNA levels were compared against each other for differential marker expression (**F**).

Bone marrow derived CD34+ hemopoietic stem cells (StemCell Technologies, Cambridge, UK) were cultured as previously described^23^. Briefly, up to 5 different donors per experimental procedure were individually expanded for 1 week for both macrophage like cell (MLC) and dendritic like cell (DLC) protocols in Stemspan SFEM II media supplemented with human serum albumin (0.05%), pen/strep, FLT3L (50 ng/ml), TPO (50 ng/ml), SR-1 (1 µM), SCF (50 ng/ml), IL6 (20 ng/ml) and IL-3 (20 ng/ml) in ultra-low attachment plates. For the second week of culture (Day 7–14), cells were replated at a density of 2 × 10^4^ cells on fresh ultra-low attachment plates and MLC and DLC protocols proceeded using SFEM II media with culture supplement combinations as described in Fig. 1A [Either Protocol 1 (MLC) or Protocol 2 (DLC)]. On Day 14, cells were replated at a density of 5 × 10^4^ cells/ml in either non tissue culture treated (NTCT) (MLC protocol) or tissue culture treated (TCT) (DLC protocol) 24 well plates in RPMI media supplemented with low IgG FBS (10%), Glutamax (2 mM) and pen/strep. Differentiation cytokines and growth factors were added at the following concentrations: IL-6 (10 ng/ml), CSF1 (50 ng/ml), CSF2 (50 ng/ml) and TGFβ (2 ng/ml) for MLC protocol differentiation, and FL3TL (100 ng/ml), CSF1 (20 ng/ml) CSF2 (20 ng/ml) and IL-4 (20 ng/ml) for DLC protocol differentiation (Fig. 1A). Cell culture media was changed every 3–4 days throughout expansion and differentiation phases. Phase contrast microscopy (100×) was used to monitor cell morphology characteristics and for image capture.

### Cell treatment, particle characterisation and cytotoxicity assessment

After differentiation, MLC or DLC cultures were treated with HDM (25 µg/ml) and DEP (25 µg/ml) alone or in combination for 24 h. Stocks of 2 mg/ml DEP (NIST Standard SRM2975) and 4 mg/ml (protein content) HDM (Stallergenes Greer, US) were prepared in sterile water and diluted accordingly in cell culture media. Corresponding diluent was used for control treated samples. Soxhlet extractions of DEP (500 mg) was conducted using 300 ml of dichloromethane in a 500 ml round-bottomed flask maintained in a temperature-controlled water bath. The DEP sample filter was extracted for 16 h, whereupon the cleaneddiesel exhaust particles (DEP.C) were removed and dried before resuspension. Prior to dilution in cell culture media, stocks of all samples were dispersed by sonication (QSonica Sonicators, CT, USA) with 4.2 × 105 kJ/m^3^. Particle size distribution was determined using nanoparticle tracking analysis (NanoSight LM10 instrument, NanoSight, Amesbury, UK) and dynamic light scattering (Zetasizer instrument, Malvern). Nanosight measurements of 25 µg/ml solutions diluted in cell culture media were assessed for a minimum of 60 s and processed using NTA 3.2 analytical software. Zetasizer measurements were carried out at 100 µg/ml solutions diluted in water. To determine necrotic and direct membrane integrity cytotoxicity, cell culture media lactate dehydrogenase (LDH) concentrations were analysed after treatment using a commercial kit (Merck; Cat# 4744926001) according to the manufacturer’s instructions. Phase contrast microscopy was carried out to evaluate morphological changes after differentiation and cell treatment protocols.

### Real time quantitative PCR

Gene expression changes between treatments were examined using PCR analysis. Cells were processed initially by lysis in RLT buffer (Qiagen) and homogenized using QIAShredder columns (Qiagen). mRNA was then purified using the RNeasy Mini Kit (Qiagen) and reverse transcribed to cDNA using a random hexamer-based protocol and Maxima reverse transcriptase (Thermofisher scientific) following the kit manufacturer’s instructions. Gene expression changes from the resulting cDNA was determined using SYBR green based real-time quantitative PCR (qRT-PCR) on the Quantstudio 6 Flex Real-Time PCR System (Applied Biosystems). Primer sequence details for each gene are detailed in Supplementary Table S1) Statistical significance compared to control values was performed using one-way ANOVA and Fisher’s LSD test in GraphPad Prism software Version 9, unless otherwise stated. Results are displayed as mean ± standard error of the mean (SEM).

### Bulk-Seq transcriptomic analysis

Global gene expression changes were determined using Poly A RNA isolated using the RNeasy Mini Kit (Qiagen). Only those samples with an RNA integrity Number (RIN) > 7.0, determined using an Agilent 2100 Bioanalyzer, proceeded to transcriptomic analysis (BGI, Hong Kong). Briefly, library construction followed by paired end (150PE) sequencing was carried out using the DNBseq platform (BGI Hong Kong). Raw sequence data were initially processed to remove adaptors, contamination, and low-quality reads. 25 million clean reads for each sample were used for further processing. Paired end reads were trimmed to remove remaining adapter and other variable sequences, followed by annotation using the Hg38 human reference genome build (REFSeq Gene ID) using CLC Genomics Workbench software (CLCBIO, Aarhus, Denmark). CLC software was also used to perform differential expression between treatment groups and results for volcano plots included Log2 fold change (FC) versus − log p values (False discovery rate). Normalised reads were also displayed as reads per kilobase per million (RPKM) and statistical comparisons between multiple treatments assessed using anova with fisher LSD test.

### Single cell sequencing

Single cell suspensions of MLC and DLC cultures were isolated, including removal of adhered cells removed using 5 mM EDTA for 30 min. Single cell sequencing was carried out using the Rhapsody platform from BD Biosciences and associated reagent kits. Single cells were initially labelled with oligonucleotide tagged antibodies to distinguish sample ID and myeloid specific markers (Abseq). All procedures were carried out using the manufacturer’s instructions. Briefly, cells were first incubated with Human BD FC block (BD) at room temperature for 10 min followed by labelling with sample tags (BD Human Single-Cell Multiplexing Kit, BD) for each sample, and BD AbSeq-Oligos (BD) for cell surface protein expression determination. This incubation was carried out for 45 min on ice. Cells were then loaded onto a microwell cartridge and lysis and transfer to capture beads was performed using the BD Rhapsody Single Cell Analysis System. Thereafter, Sample Tag, AbSeq and mRNA whole Transcriptome Analysis (WTA) libraries were prepared, indexed and sequenced using Hiseq X-ten sequencing. FASTQ files were annotated using the BD WTA Multiplex Rhapsody Analysis Pipeline Version 1.8. Single cell data was further analysed using SeqGeq version 1.7.0 (FlowJo LLC, US) software. The plug-in Lex-BDSMK was used to separate out the different samples based on sample tag ID. Cell populations were identified using Seurat 3.0 plugin and visualised using UMAP (Uniform Manifold Approximation and Projection) dimensionality reduction, based on the most highly diverse genes expressed across all cells. Differential expression between cell populations was calculated and expressed as fold change v FDR p-value significance, visualised using volcano plot.

### Immunofluorescence staining and high content imaging

After treatment, cells were collected from 24 well plates, including those adhered cells by removal with 5 mM EDTA for 30 min at 4 °C. Cells were then centrifuged using a thermos cytospin 4 (Epredia) machine onto Superfrost Microscope Slides (Epredia) at 480 rpm, for 4 min at room temperature (RT). Slides were immediately incubated 4% paraformaldehyde PBS (Sigma) for 10 min at RT followed by three 5 min washes in PBS. Cells were then permeabilised using 0.2% Triton X-100 in PBS for 15 min at RT. Cells were then washed three times with PBS for 5 min and blocked using blocking buffer (PBS containing 3% normal donkey serum + 3% FBS) for 1 h at RT. Slides were washed once in PBS followed by incubation in antibody solution (1:100 dilution in PBS containing 0.2% FBS, 0.2% normal donkey serum) for 45 min at RT or overnight at 4 °C. CD40 Antibody, anti-human, Vio^®^ Bright FITC, REAfinity™ (Cat # 130-111-067) and HLA-DR Antibody, anti-human, PE. (Cat # 130-113-964) antibodies from Miltenyi biotech (Woking, UK). After antibody incubation, slides were again washed 3 times for 5 min each with PBS. Slides were mounted with gold antifade mount solution containing DAPI for nuclear visualisation. Cell staining fluorescence intensity for each antibody stain was quantified using high content imaging ImageExpress PICO machine (Molecular Devices).

## Results

### Characterisation of CD34+ hematopoietic stem cell derived myeloid cells

To establish a panel of human primary myeloid cells, typically found within allergic and non-allergic asthmatic conditions, we used two protocols to differentiate CD34+ haematopoietic stem cells towards either macrophage like (MLC) or dendritic like cells (DLC) (Fig. 1A). Morphologically, after 21 days of culture, both populations contained adherent and non-adherent cells indicating cells of different phenotype within the cultures (Fig. 1B). Further characterisation of these cells was carried out using single cell sequencing of whole transcriptome mRNA, combined with oligonucleotide tagged antibody-based detection of cell surface protein expression (Fig. 1C–F). Cells from both protocols were combined for analysis and cell types within each protocol separated neatly upon UMAP dimensionality reduction visualisation (Left panel; Fig. 1C). Cell types within these broad populations were identified through Seurat based nearest neighbour clustering combined with manual annotation (Right panel; Fig. 1C) with the primary mRNA marker expression for each cell type displayed in Fig. 1D as a heatmap and Supplementary Fig. S1 as UMAP images. The heatmap in Fig. 1D displays expression of the cell type specific marker gene (row) for each cell (column) with the highest expression coloured yellow according to the reference bar. Cell types identified, included type 2 conventional dendritic cells (cDC2), sub-populations of macrophages (MC) and macrophage like cells (MLC) as well as M2a alternatively activated macrophages, monocytes, neutrophils, and rarer cell populations. Protein levels for myeloid markers display differences between dendritic (CD1A, CD1C) and monocyte/macrophage markers (CD14, CD16) for MLC and DLC populations respectively (Fig. 1E and Supplementary Fig. S2). The difference in whole population transcriptome between MLC and DLC cultures is also displayed from differential expression analysis of Scseq data, with typical macrophage and monocyte markers (MMP7, APOE, MARCO, CD14, SPP1 + CD163) highly expressed in MLC cultures, while Dendritic cell markers such as CD1C, CD1A, CCL17 and CCL13 highly expressed in the DLC cultures (Fig. 1F).

### Macrophage sub-populations demonstrate different responses to dust mite exposure

**Figure 2:**
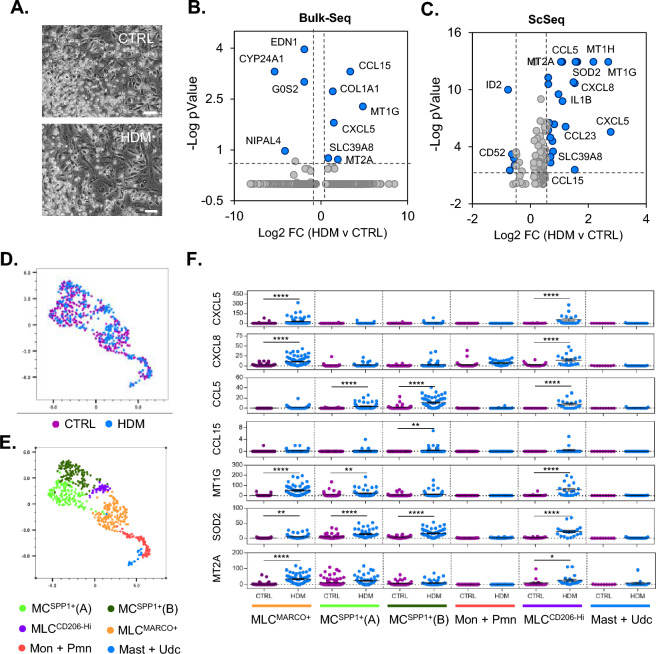
Macrophage sub-populations differentially respond to HDM exposure. Human primary CD34+ derived cells were differentiated along a myeloid trajectory towards MLC cells and treated with HDM (25 µg/ml) for 24 h. Cells were then visualised using microscopy (Scale bar; 50 µm) (**A**) and HDM transcriptional changes were examined using both bulk-seq (**B**) and Scseq (**C**) analysis. Clustering of Scseq cell populations was carried out using K-nearest neighbour (KNN) and UMAP for dimensionality reduction visualisation mapping (**D**,**E**). Both (**D**) and (**E**) panels show the same cells with different annotations. Panel (**D**) displays CTRL and HDM treated cells. Panel (**E**) displays cell type annotation including MC and MLC sub-populations. ScSeq data from each sub-population of MLC were analysed for HDM induced differential gene expression (**F**). Statistical significance was determined using ANOVA with sidaks multiple comparison post-test. (*p < 0.05, **p < 0.01, ***p < 0.001, ****p < 0.0001).

Environmental inhalation exposures including allergens such as house dust mite (HDM) can trigger asthma exacerbation events through activation of lymphoid cells such as T-cells^26^ and myeloid cells including dendritic cells^27^. Indeed, conventional dendritic cells were demonstrated to control T-cell mediated immunity in the lymph nodes, while monocyte derived dendritic cells orchestrate allergic inflammation within the lung^27^. In comparison to these populations, less is known regarding how macrophage and macrophage like cells respond to environmental stimuli such as HDM. Using the MLC culture protocol sub-populations of myeloid cells and tested their response to a single dose of HDM for 24 h. There were no obvious changes in cellular morphology (Fig. 2A) or toxicity (Supplementary Fig. S4). Examination of bulk transcriptional changes in response to HDM in these cultures revealed changes in mainly inflammatory genes such as CXCL5 and MT1G (Fig. 2B). Scseq analysis was also carried out to determine cell type specific changes in gene expression within the macrophage like cell populations (Fig. 2C–F). Differential expression analysis of the Scseq data across all cells, also identified changes in inflammatory gene expression similar to bulk-seq analysis (Fig. 2C). Additional genes were also identified, including CCL5, IL1B and CXCL8, indicating Scseq data in our study may be more sensitive to detection of transcriptional changes. To examine cell type specific changes in gene expression, the Scseq data was clustered using Seurat KNN analysis, using highly dispersed genes (with HDM differentially regulated genes removed) as input. Results are displayed as UMAP plots with each dot representing a cell (Fig. 2D,E). This resulted in even distribution of treatment across cell populations (Fig. 2D). Six main cell populations were identified on cluster analysis (Fig. 2E), which mapped closely with those sub-populations identified in Fig. 1. The most highly expressed genes in each cluster are displayed in Supplementary Tables S2–7. One of the main differences was that MC^SPP1+^ cells are subdivided in two, with MC^SPP1+^ (A) cells having higher expression of SPP1, and genes such as MMP7 and other MMP12 and MC^SPP1+^ (B) cells having higher expression APOC1, CRABP2 and GPNMB among others. Monocyte (Mon) and neutrophil (Pmn) cell populations clustered together while Mast cell and other undefined immune cells also clustered together (Fig. 2E). Representative genes within the dataset were then visualised for cell type specific regulation by HDM (Fig. 2F). Upon treatment, there was a selective induction of genes within each cell type. One of the main distinctions was the increase in CXCL5 and CXCL8 in MLC^MARCO+^ cells that was not observed in MC^SPP1+^ cells. The reverse was the case for CCL5 and CCL15. MLC^CD206-Hi^ cells, which arguably are positioned between MLC^MARCO+^ and MC^SPP1+^ cells in terms of expression of defining markers for these populations, had the largest responsiveness across the selected genes after HDM treatment (Fig. 2F). Of note, monocytes, neutrophils, mast cells and undefined immune cell populations did not display transcriptional alterations at 24 h after HDM treatment.

### Diesel exhaust particle exposure modifies dust mite responses in macrophage like cells, driven in part through Nrf2 signalling

**Figure 3:**
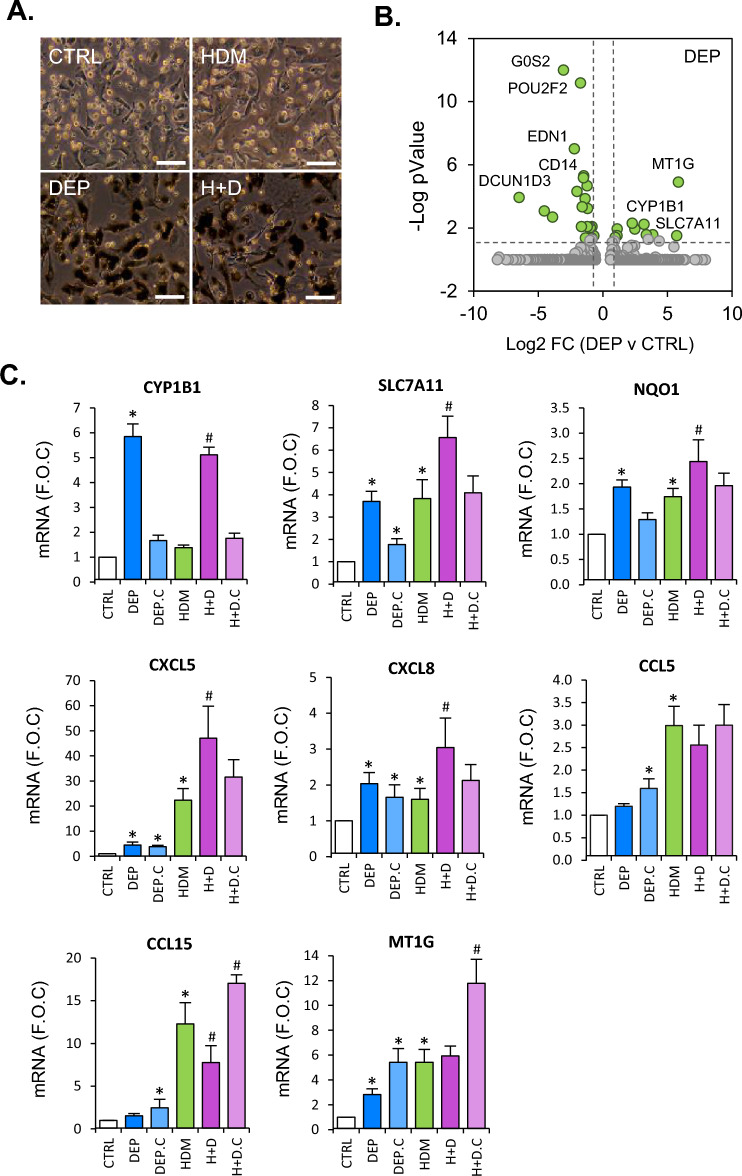
Diesel exhaust particle (DEP) exposure modifies HDM MLC responses, in part mediated by adhered DEP chemicals. Human primary CD34+ derived cells were differentiated along a myeloid trajectory towards MLC cells and treated with DEP (25 µg/ml) for 24 h. Cells were also treated with HDM (25 µg/ml) alone and in combination, with morphological assessment captured using phase contrast microscopy (Scale bar; 50 µm) (**A**). Cells were also analysed for gene expression changes in response to DEP using bulk-seq analysis (**B**). Cells were also treated with original DEP and cleaned DEP particles (DEP.C) with and without HDM (“.C” for cleaned particles) exposure all at 25 µg/ml for 24 h, and subsequently analysed for select gene expression using quantitative real time PCR (**C**). DEP.C particles had adhered chemicals removed using Soxhlet extraction. Statistical significance for PCR was carried out using anova with fisher LSD test (*p < 0.05 for exposures compared to control untreated cells. ^#^p < 0.05 for H + D and H + D.C versus HDM comparisons).

We next examined how a different type of environmental inhalation hazard, DEP impacted MLC protocol differentiated cells, either alone or in combination with HDM exposure. After 24 h of treatment, DEP alone or in combination with HDM induced a similar increase in LDH release indicating a modest level of cytotoxic effects from these particles (Supplementary Fig. S4). In contrast HDM did not significantly modify this response. Morphological assessment of cells in response to DEP revealed cellular uptake of particles, with no noticeable difference when given in combination with HDM (Fig. 3A). No significant difference in DEP particle size distribution, assessed using nanoparticle tracking analysis, was observed when compared to DEP in combination with HDM (Supplementary Fig. S5). As DEP, demonstrated significant toxicity, transcriptional differences in response to 24 h treatment were examined using bulk-seq analysis (Fig. 3B). Significant increases in a small number of genes were observed including MT1G, the aryl hydrocarbon receptor (AHR) and Nrf2 dependent gene CYP1B1^28^, and the Nrf2 dependent anti-oxidant genes SLC7A11^29^ and NQO1. Interestingly, within the MLC protocol Scseq sub-population data outlined in Fig. 2, these genes were most highly and near exclusively expressed in the MLC^CD206-Hi^ cell population (Supplementary Table S4), indicating that this cell type may be exclusively responsible for expression in DEP treated cells. This however cannot be confirmed as DEP treatments were not performed for single cell sequencing analysis. Next, we examined the effect of combined DEP and HDM on those genes identified as most highly regulated from bulk-seq data of each treatment alone (Fig. 3C). This was analysed using RT-PCR after 24 h treatment. CYP1B1 mRNA levels induced by DEP were not modified by HDM treatment. SLC7A11 and NQO1 were induced by both DEP and HDM alone and further increased upon combined DEP and HDM exposure (Fig. 3C). Similar effects were observed for CXCL5 and CXCL8 expression. For CCL5, CCL15 and MT1G genes there was a small or no effect upon DEP treatment alone, with HDM alone producing a more marked increase in expression. Combined DEP and HDM treatments did not result in a further increase in expression and for the case of CCL15 caused a significant decrease in comparison to HDM alone (Fig. 3C).

We next examined the effect of removal of chemicals found on the surface of DEP on gene expression. Treatment with cleaned DEP particles (DEP.C) completely reduced CYP1B1 expression when compared to un-cleaned DEP (Fig. 3C). Reductions in SLC7A11 and NQO1 were also observed with cleaned particles both alone and when treated in combination with HDM (H + D.C), when relevant comparisons were made with uncleaned DEP treatments. While no significant alterations were observed with DEP.C compared to DEP alone for CXCL5 and CXCL8 expression, when in combination with HDM, did not produce the enhanced expression of these genes observed when in combination with uncleaned particles. Again, in contrast to CXCL5 and CXCL8, the expression of CCL5, CCL15 and MT1G was significantly enhanced with DEP.C treatment compared to DEP both alone and when combined with HDM treatments. Dynamic light scattering analyser of particle size distribution across a larger range than possible with NTA was carried out to compare DEP and DEP.C particles (Table 1). Cleaned particles demonstrated a significantly reduced Z-average size distribution and polydispersity index (Supplementary Fig. S6).

**Table 1:** **.** Particle characterisation using dynamic light scattering.

	Z-Ave	PdI	Peak 1 size (nm)	Peak 2 size (nm)	Peak 1 area (%)	Peak 2 area (%)
DEP	229.5	0.316	254.8	4357.2	91.5	8.6
SD	17.9	0.050	29.8	745.6	7.1	7.1
DEP.C	166.5*	0.228*	189.3*	4358.9	97.6	2.4
SD	4.2	0.019	9.8	1655.8	1.5	1.5

*Indicates statistically significant difference compared to DEP particles p < 0.001. Z-Ave (Zeta average) and Peak size values (two peaks were observed on analysis) are based on intensity measurements of particle diameter.

**Figure 4:**
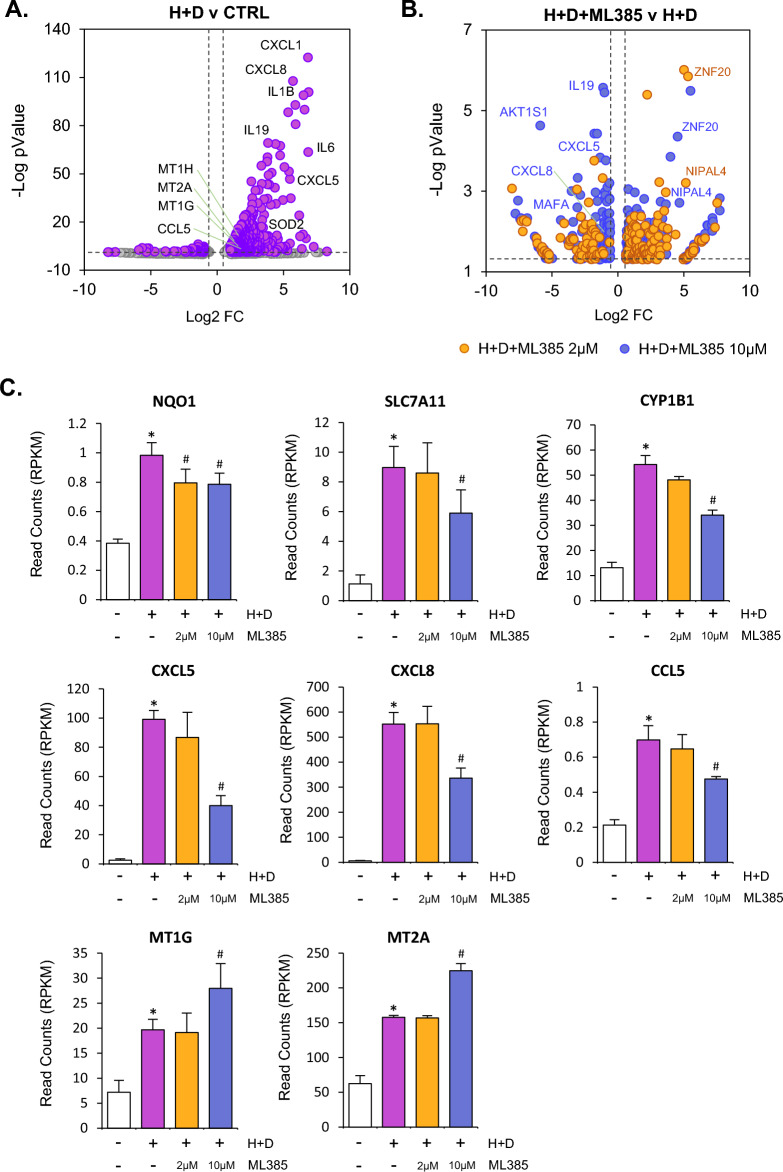
Combined HDM and DEP induced inflammatory responses are mediated through Nrf2 pathway dependent signalling. Human primary CD34+ derived cells were differentiated along a myeloid trajectory towards MLC cells and treated with combined HDM (25 µg/ml) and DEP (25 µg/ml) with or without the Nrf2 pathway inhibitor ML385 (2 µM and 10 µM) for 4 h. Cells were then analysed for gene expression changes using bulk-seq analysis (**A**–**C**). Differential expression for H + D v CTRL (**A**) and for H + D + ML385 v H + D (**B**) are displayed. Read counts (RPKM) for select genes are displayed (**C**). Statistical significance for bulk-seq RPKM comparisons in (**C**) was carried out using anova with fisher LSD test (*p < 0.05 for H + D v control untreated cells., ^#^p < 0.05 for H + D + ML385 v H + D).

The changes in Nrf2 dependent gene expression observed with these treatments, led us to examine this pathway further using the Nrf2 inhibitor ML385. This small molecule binds to the Neh1 region of Nrf2 and interferes with protein complex binding to DNA binding sequences^30^. MLC protocol differentiated cells were exposed to a combination of HDM & DEP for 4 h alone or in combination with ML385 (Fig. 4) and examined for global gene expression changes using bulk-seq analysis. 4 h was chosen to eliminate potential off-target and secondary downstream signalling events from the use of this inhibitor and to focus on immediate transcriptional events after HDM and DEP treatments. H + D treatment resulted in a large number of differentially regulated genes, with many more upregulated than downregulated genes (Fig. 4A). Upregulated genes for the most part, comprised those involved in inflammatory processes, including CXCL5, CXCL8, IL1B and IL19 (Fig. 4A). Co-treatment with ML385 resulted in an inhibition of H + D induced genes, including IL19, CXCL5 and CXCL8 (Fig. 4B). Select genes were further interrogated through visualisation of normalised count values (RPKM) across treatments (Fig. 4C). This revealed that ML385 reduced Nrf2 dependent gene expression most significantly at a 10 µM dose, indicating a functional inhibition in this pathway. Inflammatory chemokine CXCL5, CXCL8 and CCL5 expression induced by H + D was also inhibited by ML385, while metallothionein genes MT1G and MT2A were significantly upregulated (Fig. 4C).

Finally, we investigated whether dendritic like cells differentiated within our DLC protocol displayed any changes to antigen presentation pathways in response to HDM and DEP treatment. We focussed to CD40 and HLA-DRA expression changes as representative indicators of antigen presentation functionality. These genes are expressed at substantially higher levels within cDC2 cell subsets (Supplementary Figs. S1, S3A) as compared to other cell types, typical of their involvement in dendritic cell antigen presentation. DEP but not DEP.C or HDM resulted in an increase in CD40 mRNA expression (Supplementary Fig. S3B). Combined HDM + DEP increases were not dissimilar to DEP alone. Protein Levels for CD40 did not reveal any significant changes beyond a trend towards increase expression in highly positive cells with DEP treatment (Supplementary Fig. S3C). No significant changes were observed for HLADRA gene expression (Supplementary Fig. S3B) but protein levels were decreased with DEP and HDM treatment alone, which was further reduced with combined exposures (Supplementary Fig. S3C). Supplementary Figure 3D shows representative images from the high content imaging analyses, on which quantitative assessment of protein markers in Supplementary Fig. S3C were made. A focus to a greater investigation of MLC types alone was in part based on the lack of response from DLC cells to HDM.

## Discussion

In this study, we used a model of CD34 + stem cell driven MNP differentiation using two different protocols. The first protocol (DLC protocol) involved differentiation of stem cells mainly driven by the macrophage differentiation factor GMCSF^31^ and the type 2 cytokine IL-4, resulting in dendritic and type 2 inflammatory macrophage like cells. Four main cell types were identified using ScSeq analysis. Two populations of conventional type 2 dendritic cells (cDC2) were observed, one with high expression of C1QC and the chemokines CCL18, CCL26 and CCL23 (cDC2^C1qCHi^), while the other population had higher expression of CD1A and CCL17 (cDC2^CD1A+^). cDC2 cells contribute to allergic airway disease development through antigen capture, presentation, and direction of adaptive immunity. They have also been identified as unique among dendritic cell subtypes, as increased in human allergic asthmatic airways on acute dust mite exposure compared to allergic non-asthmatic control patients^12^. Similarity of both cDC2 populations in our study to MAC2 populations identified in this latter study by Alladina et al., was noted^12^, although levels of cDC2 markers in these MAC2 cells were much lower, perhaps indicating a transitional sub-population on the trajectory to cDC2 differentiation. CCL22 expressing cells were also identified within the DLC protocol, also a typical marker of cDC2. However, these cells (MC^CCL22+^) did not express CD1C and had higher expression of FABP4 indicating macrophage type cells, which corelated with MAC1 FABP4 positive cells found within asthmatic airways^12^. Finally, CD206+, FCER2 Hi, CD1C negative population of cells, indicative of M2a alternatively activated macrophages (AAM) were also identified and again have been observed within allergic airway disease states^32,33^.

We tested cells within the DLC protocol for their responsiveness to HDM and DEP treatment. We observed the DC maturation marker CD40 gene expression as increased upon DEP exposure but not significantly modified by HDM. Similar results were observed with protein expression detection of CD40, although the low number of positive cells, indicate that this response was not a significant part of the overall response of the DLC population. Alternatively, HLA-DRA expression was downregulated on exposure to DEP and HDM, indicating that antigen presentation capabilities of these cells may be compromised by exposure to these environmental toxins. While these may be interesting observations to explore in future work, it was decided to focus on macrophage cell subtypes identified within the MLC protocol to examine more detailed genomic responses to HDM and DEP and to corelate this with cells found within asthmatic and allergic airway disease.

The MLC protocol was found to generate a range of different MNP cell types upon ScSeq analysis, but also a small numbers of other myeloid cells such as neutrophils. One of these MNP cell types was identified as macrophages with a high expression of osteopontin (SPP1), APOE and CHIL3L1 and labelled MC^SPP1+^. SPP1+ macrophages have been identified previously as increased in fibrotic tissues and playing a role in the pulmonary fibrosis and resolution of inflammation^34,35^. A subset of macrophages expressing SPP1, GPNMB, FABP5, and CD63 have also recently been identified in pulmonary and hepatic fibrosis associated with scar formation^35^. In addition to fibrotic environments, these MC^SPP1+^ cells are observed within asthmatic airways as pathogenic monocyte derived cells, specifically identified as the MC2 population in this article^12^. CD206 (MRC1) has been recognised as a marker of the M2 phenotype of macrophages associated with tissue repair and type 2 inflammatory environments^36^. These alternatively activated macrophages (AAM) are highly expressed in asthmatic airways^21^, where they are thought to play a pathogenic role in airway disease. We identified a small population of these cells within our dataset termed MLC^CD206Hi^. These cells differ from CD206 expressing AAM identified in the DLC culture protocol as they have high levels of CCL7, CD14 and CD163 indicative of immature monocyte derived AAM and thus labelled macrophage like cells. A further sub-population of macrophages characterised by high expression of the M2 marker CD163, C1qC and another scavenger receptor MARCO was identified within our dataset (MLC^MARCO+^). These cells identified previously as Mac1 cells in the study by Alladina et al., were present within the airways of allergic and asthmatic patients^12^. An additional population of CD14 + cells was identified as highly expressing MCEMP1 and HP indicating classical monocytes^37,38^. Neutrophils (Pmn) expressing MPO, BPI and LTF and Mast cells expressing CPA3 were also identified within the MLC protocol. Despite the identification of these cell types within human allergic airway disease, the primary response of these cells to environmental exposures has not been characterised in any great detail.

Exposure of MLC protocol differentiated cells to HDM for 24 h revealed an increase in gene expression of mainly inflammatory mediators in our study. MC^SPP1+^, MLC^CD206Hi^ and MLC^MARCO+^ cells all responded to HDM exposure, while monocytes, neutrophils, mast cells and undefined cells did not. Of particular interest was the difference between cell types that did respond, with MC^SPP1+^. cells displaying increased CXCL5 and CXCL8 expression while MLC^MARCO+^ showing no change in their expression. The reverse was true for these two cell types when examining CCL5 and CCL15 responses. There are two main categories of chemokines CXC and CC, which have their own general broad groups of cell types that they attract, albeit with some small overlap. CXC chemokines primarily attract neutrophils as a first line defence against tissue injury and microbial infection^39^ and typically form part of an acute or innate immune response. CC chemokines on the other hand recruit mainly monocytes and lymphocytes, which in addition to some innate functions, mount adaptive immune responses such as T-cell driven type 2 immunity as observed in allergic airway disease^39–41^. Indeed, MLC^MARCO+^ cells have been identified as tolerogenic macrophages^42^ or a sub-type of alternatively activated macrophages (AAM) found within type 2 allergic conditions^43^ and therefore fits that these cells would selectively produce chemokines involved in lymphocyte mediated adaptive immune processes. Specifically, CCL15 has been identified as a potent chemoattractant for monocytes^40^ and more recently from eosinophils and to promote type 2 airway inflammation^40,41^. CCL5 on the other hand, appears to have a role in both T1 and T2 adaptive immune responses^44^. The role of these cells within an inflammatory tissue environment and the consequences for how they respond to allergen exposures such as HDM, is a complex issue. However, the identification that different cell types can selectively respond, reveals a capacity for a high degree of nuance tissue responses where different cell macrophage and myeloid cell populations are present. Indeed, as described previously up to 14 different types of MNPs are present with human allergic and asthmatic airways, where exposures to inhaled material occurs. It is likely the spatial distribution of immune cells and interaction with surrounding structural cell types within the airway compartment governing local inflammatory microenvironments that influence the proportion of each cell type present in health and disease. This coupled with observation that different cell types can respond differently to HDM exposure could be suggested as a way in which different immune responses and for chemokine expression, appropriate immune cells are recruited to areas where a specific need is required at a local microenvironmental level, whether it be from naïve exposure to a repeated exposure in an already inflammatory tissue. This suggestion, while possible has not been determined so far. With the advent of further studies and techniques such as spatial transcriptomics, such information on the reason for differential myeloid cell responsiveness may become clearer.

We next investigated in this study, the influence of DEP, another inhaled toxic exposure implicated in causing detrimental effects in asthma and allergic airway disease. We did not have the resources to explore responses of individual cell type responses to DEP exposure as was carried out for HDM using single cell sequencing, but rather focussed on using RT-PCR and bulk-seq transcriptome analysis to determine how these MNP populations responded to DEP alone and when combined with HDM exposure. Some preliminary observations of HDM and DEP exposure effects in DLC protocol generated MNPs did not reveal any significant activation of dendritic cell antigen presentation function and perhaps revealed some diminished effects. Again, due to resource limitation we focussed the majority of the analysis of DEP effects using MLC protocol generated population of cells. Bulk RNA-seq analysis of MLC cells exposed to DEP revealed changes in gene expression consistent with previous understanding of how these particles interact with cells to cause toxic effect^45–47^. A large increase in CYP1B1 gene expression, a gene dependent on the aryl hydrocarbon receptor for inducible expression was observed. DEPs are typically coated with polycyclic aromatic hydrocarbons such as BAP, known ligands for the AHR and therefore CYP1B1 gene expression would be expected upon DEP exposure, and the results indicate that these particles used in this study may elicit their toxic effects through this pathway. To further delineate the relative contribution of physical particle versus toxic chemical effects, DEPs were cleaned of their chemicals using Soxhlet extraction. These cleaned particles (DEP.C) did not elicit a CYP1B1 response as expected. A small reduction in particle size properties was observed with DEP.C compared to DEP. It is possible these changes influence toxicity outcomes in these cells but given small change in size and the reduction of AHR dependent gene expression specific to chemical presence on the particles, it is more likely differences can be attributed to the absence of chemicals on the DEP.C particles. SLC7A11 and NQO1, are genes induced by activation of the oxidant stress responsive transcription factor Nrf2. Their expression was induced by both DEP and HDM and increased further with combined exposure. Similar to CYP1B1 expression these oxidative stress gene responsiveness with DEP exposure was diminished when adsorbed chemicals were removed. Interestingly, the response of different chemokines didn’t necessarily follow this pattern. For example, CXCL5 and CXCL8 expression of combined H + D exposure was diminished when DEP.C cleaned particles were used instead of DEP for combined exposures. In opposition to this, for CCL5 and CCL15, the cleaned particles produced a much higher level of expression in the presence of HDM. Based on our understanding of how different cell types respond to HDM in the production of these cytokines from our ScSeq results, we can speculate that these same cell types may also have selectivity for how they respond to chemical and pollutant particle exposure. This level of nuanced behaviour of MNPs is likely necessary to direct appropriate immune responses to toxic exposures dependent on the chemical and physical nature of the pollutant. Further speculation on the exact nature of these directed responses may not be informative without further evidence of the molecular and cellular events involved in more complex tissue environments.

We can however give some insight into one aspect of the molecular response to how DEP modified HDM exposure responses from our observations with the Nrf2 inhibitor ML385. Chemokine gene expression induced by H + D exposure was reduced by ML385 treatment, indicating Nrf2 and by inference oxidative stress in the transcriptional response of genes such as CXCL5 and CCL5. The timepoint of 4 h treatment was used to minimise the impact of secondary signalling events due to the action of Nrf2 dependent induction of antioxidant genes, and therefore we suggest that this is a direct action of Nrf2 on gene regulation, rather than any mitigation of oxidative stress signal from de nova upregulation of anti-oxidant proteins. It is clear that additional transcription signalling events are likely to also play a role in how DEP modifies HDM inflammatory gene expression. Indeed, activation of the AHR can lead to changes in NF-kB dependent gene expression through direct association between the two transcription factors and induction of gene expression^48^. Interestingly, previous investigation into the signalling events involved in how PAH pollutants regulate chemokine gene expression in airway epithelial cells demonstrated that different AHR ligands, as found on DEP particles, can induce CXCL8 and CCL5 but that only CXCL8 responses were dependent on the transcription factor NF-kB^49^. This differential dependence on NF-kB signalling may well underly the reasons why CXCL and CCL chemokines were differentially regulated by DEP in MLCs in our study.

An additional observation of note in our study, was the increase of metallothionein genes such as MT1G with Nrf2 inhibition. Metallothioneins have been observed to respond to changes in soluble metals including toxic metal exposures^50^. They have also been suggested as inhibitors of NF-kB induced inflammatory gene expression including as a counterbalance to temper inflammatory signals to prevent excessive responses to inflammatory stimuli^51–54^. The similarity of Nrf2 inhibition responses including increased MT1G expression to changes observed with removal of toxic adhered chemicals from DEP would support the notion that these pollution DEP chemicals have at least part of their biological effect through oxidative or electrophilic stress. It is unlikely that these changes in metallothionein gene expression are related to toxic metals on the particles, given that cleaning and washing the particles in solvent, which removes soluble material resulted in an increase in MT1G expression. Therefore, it is more likely, regulated expression is perhaps secondary to changes in oxidative stress levels within the cells. No clear direct responsiveness of metallothionein (MT) to Nrf2 has been observed to date in the literature. Metallothionein expressing macrophages have however been identified within the lung and have been suggested to play a role in tissue defence, repair and homeostasis^55,56^. In summary, we have demonstrated selective gene expression induced by HDM, which is dependent on the type of MNP phenotype. MLC^CD206Hi^ in particular are likely to play a prominent role as primary responders to inhaled HDM exposure during active inflammation as these cells displayed the greatest response. These cells have been identified in allergic inflammatory tissue as AAMs, located within airways of asthmatic patients to respond and direct immune responses accordingly. The impact of DEP on HDM inflammatory effects was also observed and likely a consequence of Nrf2 pathway activation and changes in oxidative stress levels within the cells. Ultimately this study highlights how environmental inhaled exposures may influence cell inflammatory events at a molecular level dependent on the original cell phenotype and that these should be considerations when modelling such events in vitro. We also want to highlight that the results in this study arise from in vitro models and exposure methods, which may not reflect exactly what occurs within human disease conditions. By using in depth cell type characterisation with ScSeq and identifying some of the same cell types in active allergic asthmatic lung, we have confidence our test system has the capability to accurately model at least some of the cellular responses and mechanisms relevant for human disease. We believe this work, while not capable of capturing the myriad of cellular responses and interactions in complex inflammatory disease, it does bring a more detailed understanding of how different MNP subtypes respond and potentially propagate disease. It is interesting to speculate how targeting particular subtypes in disease to remove these propagating chemokine signal would impact disease. It is however beyond the remit of this study to test this hypothesis.

### Supplementary Information

Supplementary Information 1.

Supplementary Information 2.

Supplementary Information 3.

## Data Availability

The datasets used and/or analysed during the current study are available from the corresponding author on reasonable request.

## Abbreviations

MNP: Mononuclear phagocyte
ScSeq: Single cell sequencing
AAM: Alternatively activated macrophages
CTRL: Control
DC: Dendritic cell
DLC: Dendritic like cell
MC: Macrophage cell
MLC: Macrophage like cell
FC: Fold change
F.O.C: Fold over control
HDM: House dust mite
DEP: Diesel exhaust particles
DEP.C: Cleaned diesel exhaust particles
H + D: House dust mite combined with diesel exhaust particles
LDH: Lactate dehydrogenase
SEM: Standard error of the mean
UMAP: Uniform manifold approximation and projection
WTA: Whole transcriptome analysis
FDR: False discovery rate
cDC2: Type 2 conventional dendritic cells
MT: Metallothionein
