# Supplementary material for: Mononuclear phagocyte sub-types in vitro display diverse transcriptional responses to dust mite exposure
Source: Sci Rep. 2024 Jun 20;14:14187. doi: 10.1038/s41598-024-64783-1 (PMC11189906; doi:10.1038/s41598-024-64783-1)
Supplement: Supplementary file 1 — Supplementary Information 1. [file 41598_2024_64783_MOESM1_ESM.pptx]

## Slide 1
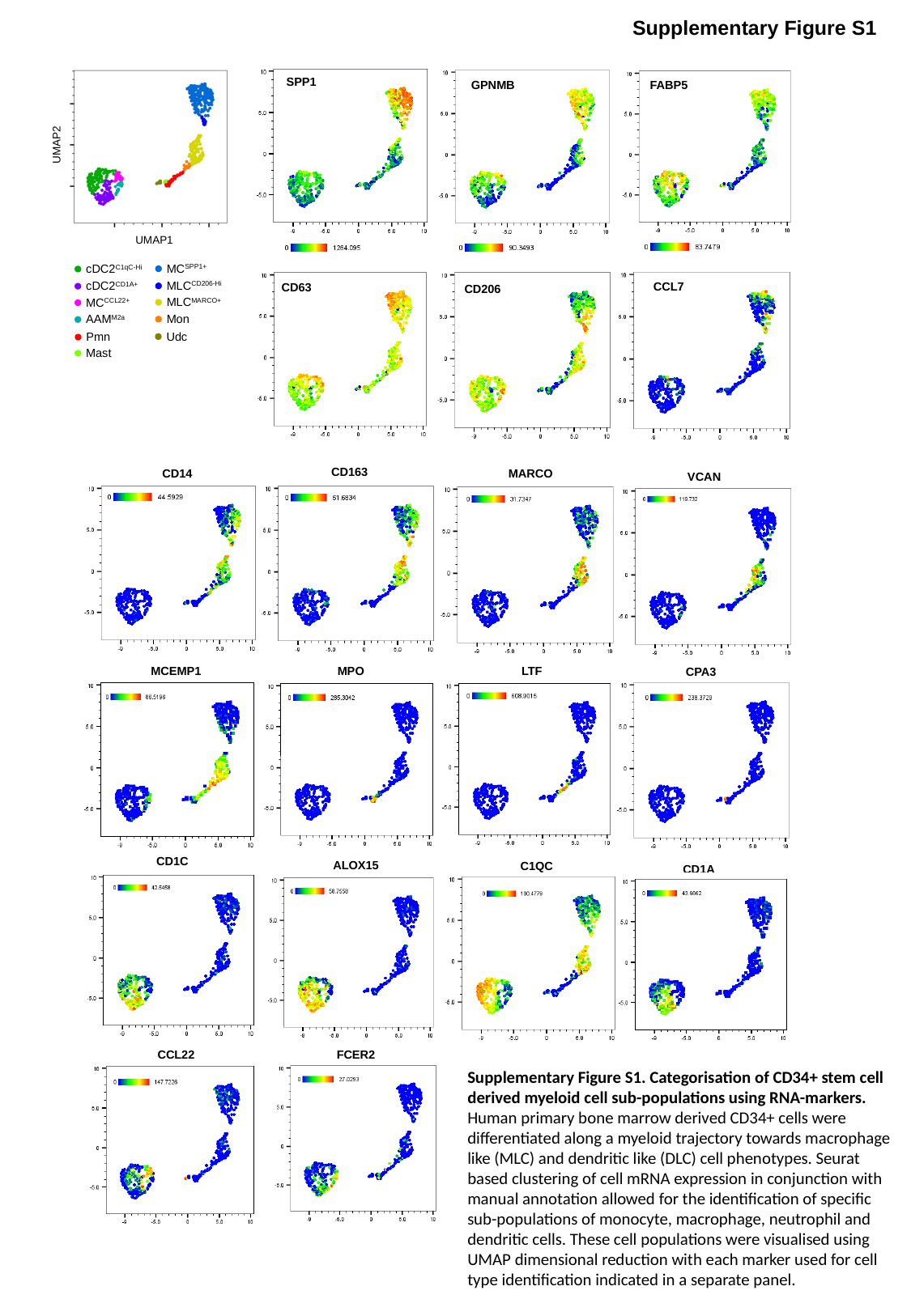

Supplementary Figure S1
SPP1
GPNMB
FABP5
UMAP2
UMAP1
MCSPP1+
cDC2C1qC-Hi
MLCCD206-Hi
cDC2CD1A+
MLCMARCO+
MCCCL22+
Mon
AAMM2a
Udc
Pmn
Mast
CD206
CCL7
CD63
CD163
CD14
MARCO
VCAN
MCEMP1
MPO
LTF
CPA3
CD1C
ALOX15
C1QC
CD1A
CCL22
FCER2
Supplementary Figure S1. Categorisation of CD34+ stem cell derived myeloid cell sub-populations using RNA-markers. Human primary bone marrow derived CD34+ cells were differentiated along a myeloid trajectory towards macrophage like (MLC) and dendritic like (DLC) cell phenotypes. Seurat based clustering of cell mRNA expression in conjunction with manual annotation allowed for the identification of specific sub-populations of monocyte, macrophage, neutrophil and dendritic cells. These cell populations were visualised using UMAP dimensional reduction with each marker used for cell type identification indicated in a separate panel.

## Slide 2
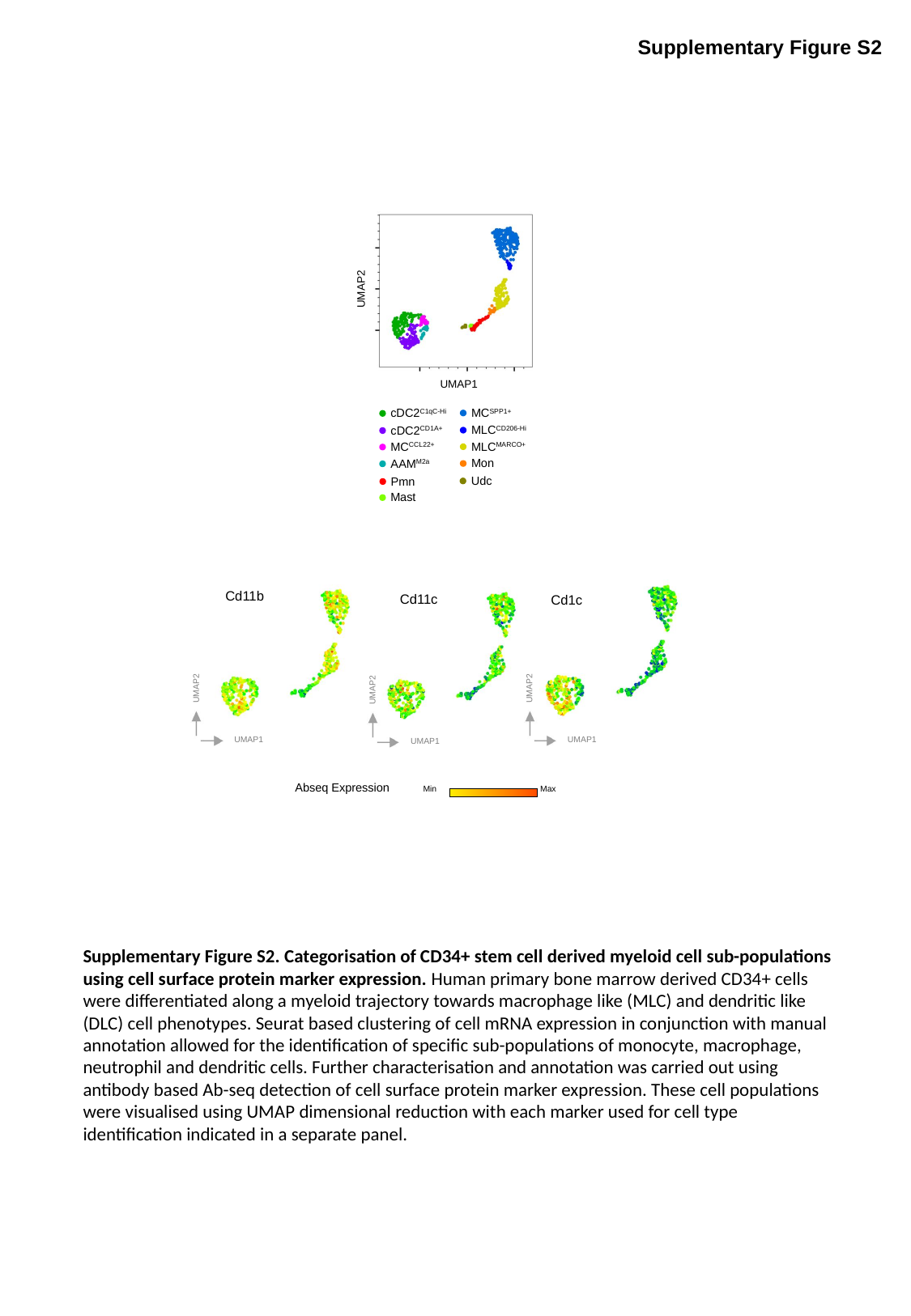

Supplementary Figure S2
UMAP2
UMAP1
MCSPP1+
cDC2C1qC-Hi
MLCCD206-Hi
cDC2CD1A+
MLCMARCO+
MCCCL22+
Mon
AAMM2a
Udc
Pmn
Mast
Cd11b
Cd11c
Cd1c
UMAP2
UMAP1
UMAP2
UMAP1
UMAP2
UMAP1
Abseq Expression
Min
Max
Supplementary Figure S2. Categorisation of CD34+ stem cell derived myeloid cell sub-populations using cell surface protein marker expression. Human primary bone marrow derived CD34+ cells were differentiated along a myeloid trajectory towards macrophage like (MLC) and dendritic like (DLC) cell phenotypes. Seurat based clustering of cell mRNA expression in conjunction with manual annotation allowed for the identification of specific sub-populations of monocyte, macrophage, neutrophil and dendritic cells. Further characterisation and annotation was carried out using antibody based Ab-seq detection of cell surface protein marker expression. These cell populations were visualised using UMAP dimensional reduction with each marker used for cell type identification indicated in a separate panel.

## Slide 3
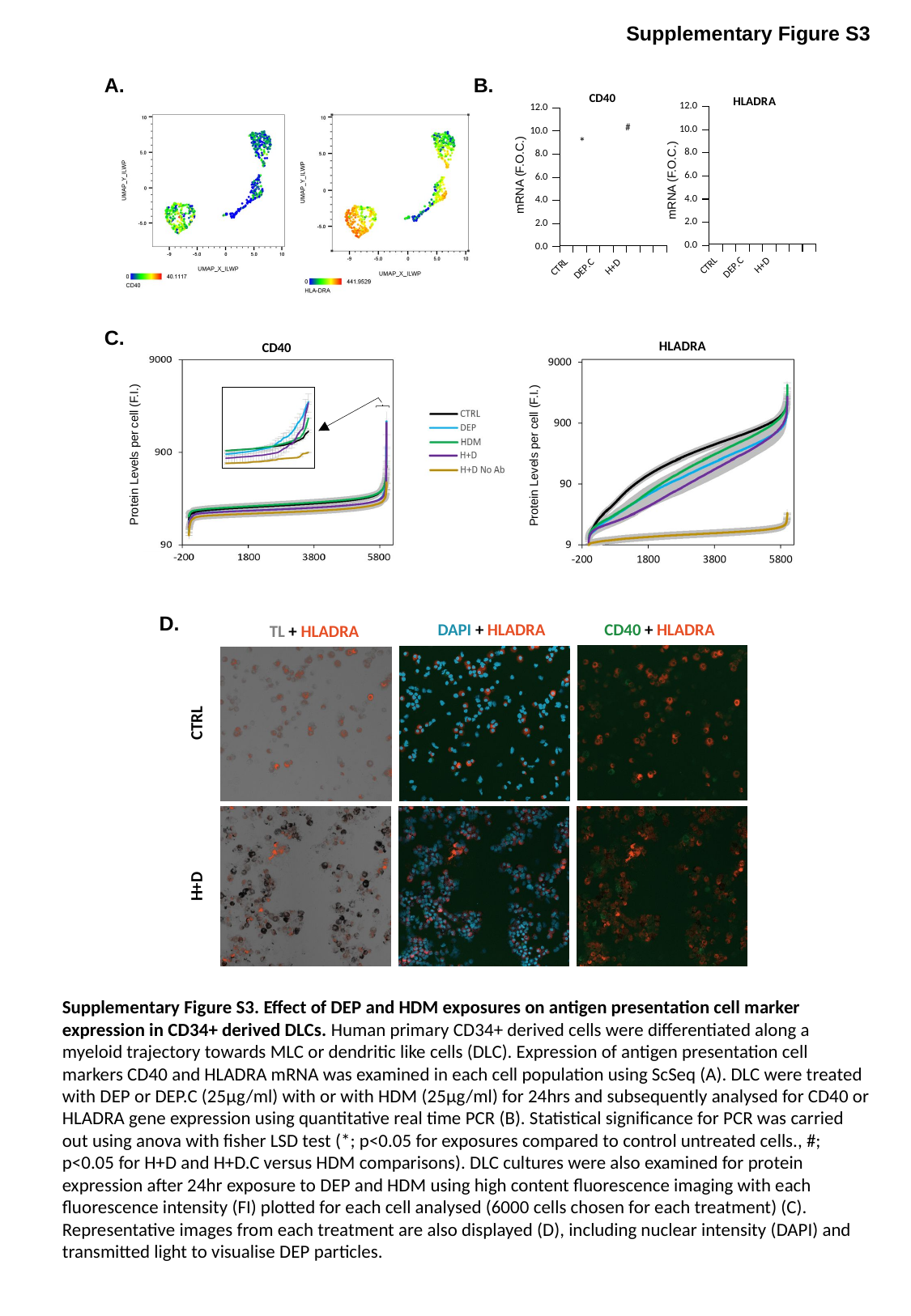

Supplementary Figure S3
### Chart: HLADRA
| Category | |
|---|---|
| CTRL | 1.0 |
| DEP | 1.038514982871081 |
| DEP.C | 0.9706816796650383 |
| HDM | 0.8653690588426187 |
| H+D | 0.907438176121163 |
| H+D.C | 0.9318356798783144 |
### Chart: CD40
| Category | |
|---|---|
| CTRL | 1.0 |
| DEP | 1.5217139842414276 |
| DEP.C | 1.15481053863906 |
| HDM | 1.1752004798366875 |
| H+D | 1.5426224048427137 |
| H+D.C | 1.2353861789886702 |mRNA (F.O.C.)
A.
B.
C.
HLADRA
Protein Levels per cell (F.I.)
CD40
Protein Levels per cell (F.I.)
D.
CD40 + HLADRA
DAPI + HLADRA
TL + HLADRA
CTRL
H+D
#
*
mRNA (F.O.C.)
Supplementary Figure S3. Effect of DEP and HDM exposures on antigen presentation cell marker expression in CD34+ derived DLCs. Human primary CD34+ derived cells were differentiated along a myeloid trajectory towards MLC or dendritic like cells (DLC). Expression of antigen presentation cell markers CD40 and HLADRA mRNA was examined in each cell population using ScSeq (A). DLC were treated with DEP or DEP.C (25µg/ml) with or with HDM (25µg/ml) for 24hrs and subsequently analysed for CD40 or HLADRA gene expression using quantitative real time PCR (B). Statistical significance for PCR was carried out using anova with fisher LSD test (*; p<0.05 for exposures compared to control untreated cells., #; p<0.05 for H+D and H+D.C versus HDM comparisons). DLC cultures were also examined for protein expression after 24hr exposure to DEP and HDM using high content fluorescence imaging with each fluorescence intensity (FI) plotted for each cell analysed (6000 cells chosen for each treatment) (C). Representative images from each treatment are also displayed (D), including nuclear intensity (DAPI) and transmitted light to visualise DEP particles.

## Slide 4
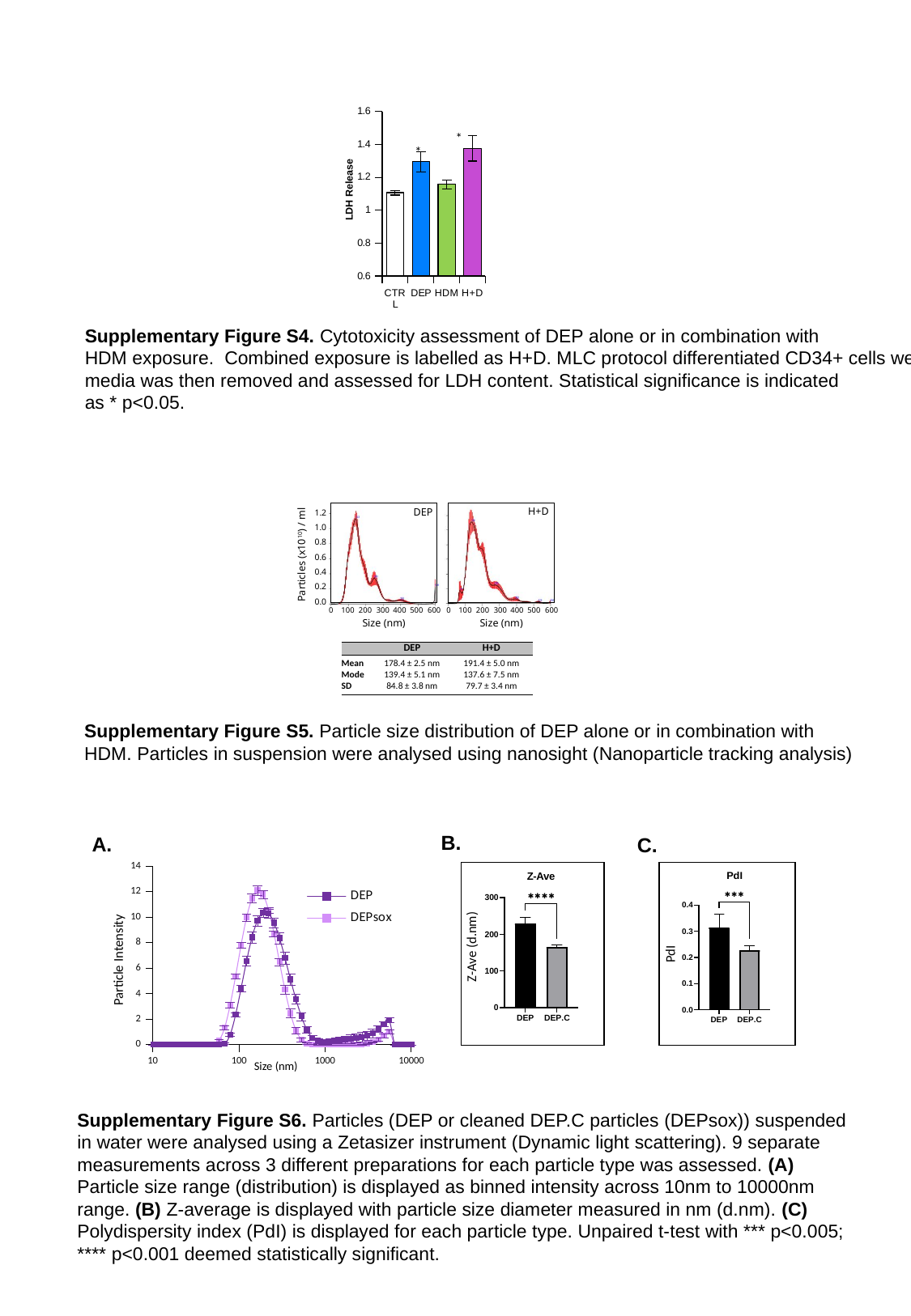

### Chart
| Category | |
|---|---|
| CTRL | 1.1067786690950627 |
| DEP | 1.2946061940433515 |
| HDM | 1.1564235787606663 |
| H+D | 1.3767287932609182 |LDH Release
*
*
Supplementary Figure S4. Cytotoxicity assessment of DEP alone or in combination with
HDM exposure. Combined exposure is labelled as H+D. MLC protocol differentiated CD34+ cells were treated for 24hrs, cell culture
media was then removed and assessed for LDH content. Statistical significance is indicated
as * p<0.05.
DEP
1.2
1.0
0.8
Particles (x1010) / ml
0.6
0.4
0.2
0.0
0
100
200
300
400
500
600
Size (nm)
H+D
0
100
200
300
400
500
600
Size (nm)
| | DEP | H+D |
| --- | --- | --- |
| | | |
| Mean | 178.4 ± 2.5 nm | 191.4 ± 5.0 nm |
| Mode | 139.4 ± 5.1 nm | 137.6 ± 7.5 nm |
| SD | 84.8 ± 3.8 nm | 79.7 ± 3.4 nm |
| | | |
Supplementary Figure S5. Particle size distribution of DEP alone or in combination with
HDM. Particles in suspension were analysed using nanosight (Nanoparticle tracking analysis)
B.
A.
C.
### Chart
| Category | DEP | DEPsox |
|---|---|---|Particle Intensity
Size (nm)
Z-Ave (d.nm)
PdI
Supplementary Figure S6. Particles (DEP or cleaned DEP.C particles (DEPsox)) suspended in water were analysed using a Zetasizer instrument (Dynamic light scattering). 9 separate measurements across 3 different preparations for each particle type was assessed. (A) Particle size range (distribution) is displayed as binned intensity across 10nm to 10000nm range. (B) Z-average is displayed with particle size diameter measured in nm (d.nm). (C) Polydispersity index (PdI) is displayed for each particle type. Unpaired t-test with *** p<0.005; **** p<0.001 deemed statistically significant.
