# Supplementary material for: Mononuclear phagocyte sub-types in vitro display diverse transcriptional responses to dust mite exposure
Source: Sci Rep. 2024 Jun 20;14:14187. doi: 10.1038/s41598-024-64783-1 (PMC11189906; doi:10.1038/s41598-024-64783-1)
Supplement: Supplementary file 2 — Supplementary Information 2. [file 41598_2024_64783_MOESM2_ESM.pptx]

## Slide 1
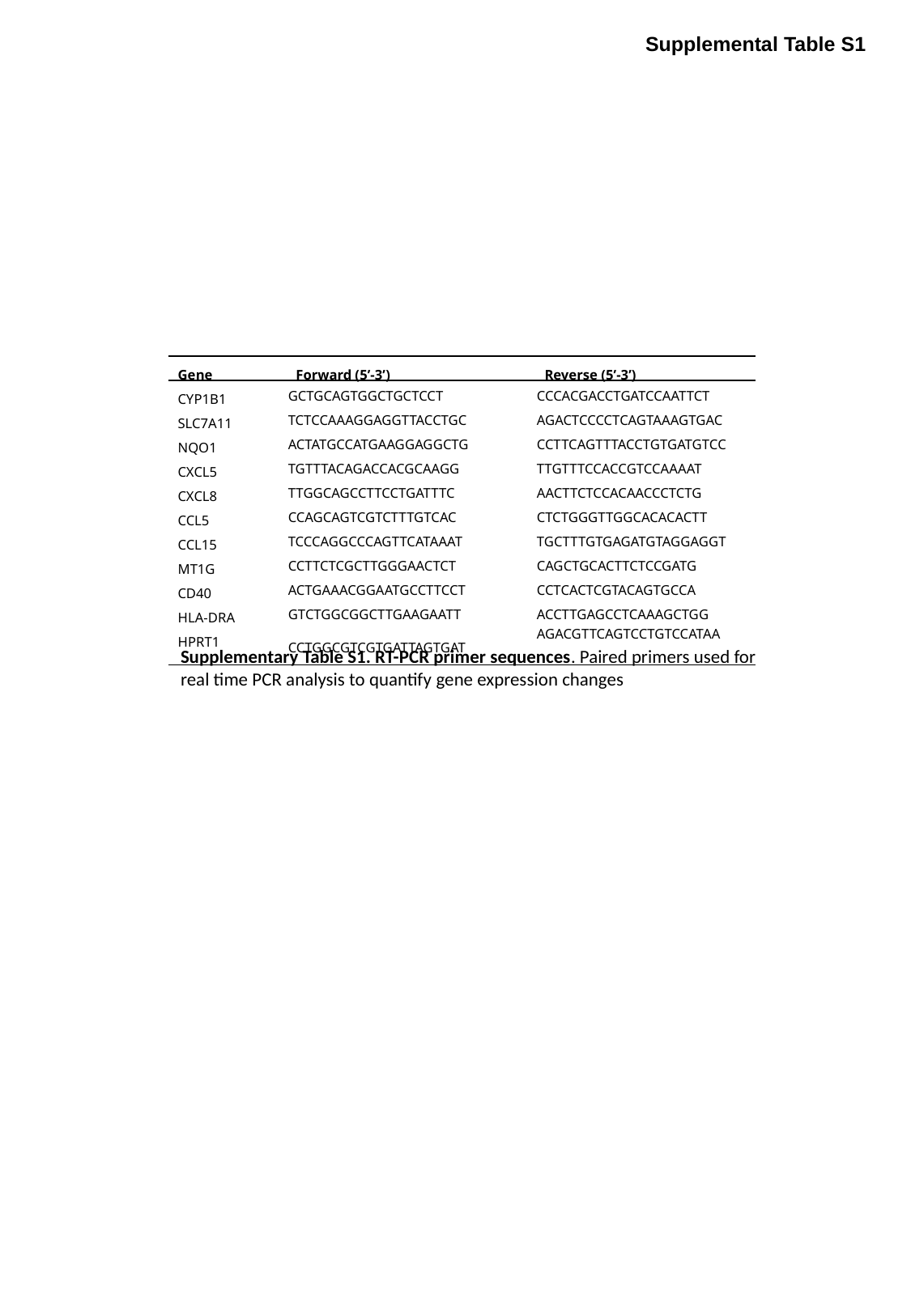

Supplemental Table S1
| Gene | Forward (5’-3’) | Reverse (5’-3’) |
| --- | --- | --- |
| CYP1B1 | GCTGCAGTGGCTGCTCCT | CCCACGACCTGATCCAATTCT |
| SLC7A11 | TCTCCAAAGGAGGTTACCTGC | AGACTCCCCTCAGTAAAGTGAC |
| NQO1 | ACTATGCCATGAAGGAGGCTG | CCTTCAGTTTACCTGTGATGTCC |
| CXCL5 | TGTTTACAGACCACGCAAGG | TTGTTTCCACCGTCCAAAAT |
| CXCL8 | TTGGCAGCCTTCCTGATTTC | AACTTCTCCACAACCCTCTG |
| CCL5 | CCAGCAGTCGTCTTTGTCAC | CTCTGGGTTGGCACACACTT |
| CCL15 | TCCCAGGCCCAGTTCATAAAT | TGCTTTGTGAGATGTAGGAGGT |
| MT1G | CCTTCTCGCTTGGGAACTCT | CAGCTGCACTTCTCCGATG |
| CD40 | ACTGAAACGGAATGCCTTCCT | CCTCACTCGTACAGTGCCA |
| HLA-DRA | GTCTGGCGGCTTGAAGAATT | ACCTTGAGCCTCAAAGCTGG |
| HPRT1 | CCTGGCGTCGTGATTAGTGAT | AGACGTTCAGTCCTGTCCATAA |
| | | |
Supplementary Table S1. RT-PCR primer sequences. Paired primers used for real time PCR analysis to quantify gene expression changes
